# Cardiovascular Risk Assessment by SCORE2 Predicts Risk for Colorectal Neoplasia and Tumor-Related Mortality

**DOI:** 10.3390/jpm12050848

**Published:** 2022-05-23

**Authors:** Sarah Wernly, Georg Semmler, Andreas Völkerer, Richard Rezar, Leonora Datz, Konrad Radzikowski, Felix Stickel, Elmar Aigner, David Niederseer, Bernhard Wernly, Christian Datz

**Affiliations:** 1 Department of Internal Medicine, General Hospital Oberndorf, Teaching Hospital of the Paracelsus Medical University Salzburg, 5020 Salzburg, Austria; sarah@wernly.net (S.W.); a.voelkerer@kh-oberndorf.at (A.V.); leo_datz@hotmail.com (L.D.); k.radzikowski@kh-oberndorf.at (K.R.); bernhar@wernly.net (B.W.); 2 Department of Internal Medicine III, Division of Gastroenterology and Hepatology, Medical University of Vienna, 1090 Vienna, Austria; georg.semmler@hotmail.com; 3 Clinic of Internal Medicine II, Department of Cardiology, Paracelsus Medical University of Salzburg, 1090 Salzburg, Austria; r.rezar@salk.at; 4 Department of Cardiology, University Hospital Zurich, 8091 Zurich, Switzerland; felix.stickel@uzh.ch; 5 Department of Gastroenterology and Hepatology, University Hospital of Zurich, 8091 Zurich, Switzerland; e.aigner@salk.at; 6 First Department of Medicine, Paracelsus Medical University Salzburg, 5020 Salzburg, Austria; david.niederseer@uzh.ch; 7 Institute of General Practice, Family Medicine and Preventive Medicine, Paracelsus Medical University, 5020 Salzburg, Austria

**Keywords:** primary prevention, risk assessment, colorectal adenoma and carcinoma, cancer screening, risk score

## Abstract

Objectives: The European Society of Cardiology endorsed SCORE2 to assess cardiovascular risk. The aim of this observational, retrospective study was to assess whether SCORE2 is associated with colorectal neoplasia in an asymptomatic screening population. Further, we evaluated if SCORE2 predicts tumor-related mortality. Methods: We included 3408 asymptomatic patients who underwent a screening colonoscopy. We calculated SCORE2 for each participant and stratified patients according to their predicted 10-year risk of cardiovascular disease: SCORE2 0–4.9%, SCORE2 5–9.9%, and SCORE2 ≥ 10%. We assessed the association between SCORE2 as a continuous variable, the presence of colorectal neoplasia using multilevel logistic regression, and SCORE2 and mortality using Cox regression. Results: In total, 1537 patients had a SCORE2 of 0–4.9%, 1235 a SCORE2 of 5–9.9%, and 636 a SCORE2 ≥ 10%. The respective rates of colorectal neoplasia were 20%, 37%, and 44%. SCORE2 was associated with the presence of any (OR 1.11 95%CI 1.09–1.12; *p* < 0.001) and advanced colorectal neoplasia (OR 1.06 95%CI 1.08–1.13; *p* < 0.001) in univariate analysis. After multivariable adjustment (age, sex, family history, and metabolic syndrome) a higher SCORE2 remained associated with higher odds for any (aOR 1.04 95%CI 1.02–1.06; *p* = 0.001) and advanced (aOR 1.06 95%CI 1.03–1.10; *p* < 0.001) colorectal neoplasia. SCORE2 was associated with both all-cause (HR 1.11 95%CI 1.09–1.14; *p* < 0.001) and tumor-related mortality (HR 1.10 95%CI 1.05–1.14; *p* < 0.001). Conclusions: We found that SCORE2 is associated with the presence of colorectal neoplasia. Clinicians could kill two birds with one stone calculating SCORE2. In patients with a high SCORE2, screening colonoscopy aside from cardiovascular risk mitigation could improve outcomes.

## 1. Introduction

Contrary to the idiom that there is no glory in prevention, we think that prevention of disease is the noblest form of medicine. Investing in prevention is also worthwhile from a health economic perspective. The prevention of two long lasting pandemics, cardiovascular and oncological diseases, is particularly urgent [1]. Additionally, cardiovascular diseases and colorectal neoplasms share similar risk factors and pathophysiological mechanisms [2], and cardiovascular risk is associated with a higher risk for colorectal neoplasia [3].

Colorectal cancer (CRC) screening is an effective strategy to improve outcomes and reduce mortality [4]. However, the participation rates for CRC screening after invitation vary from 25% for colonoscopy screening to 39% for the fecal immunochemical test [5] and might miss patients at highest risk. Measures to increase these rates are therefore needed.

Although neither systematic nor opportunistic screening for cardiovascular disease has been shown to reduce mortality in healthy individuals, screening for cardiovascular risk factors is often requested by patients and recommended in the prevention guideline of the European Society of Cardiology (ESC) [6]. Several easily accessible scores help to calculate the 10-year risk of fatal and non-fatal cardiovascular disease. The SCORE2 risk prediction model was recently introduced by the ESC [7]. This score is a sex- and country-specific score that estimates CVD risk according to age, sex, smoking history, systolic blood pressure, and cholesterol levels.

Given the common risk factors of cardiovascular disease and CRC, it is possible that SCORE2 predicts both risks. Since SCORE2 is expected to soon be used in clinical practice worldwide, such an association would be of great importance in clinical practice. This association could further remind patients and doctors of the relevance of CRC screening that might otherwise have been forgotten or repressed.

To date, there is limited data on the potential of cardiovascular risk scores to predict colorectal adenomas. Most existing studies deal with Asian collectives [2]. Therefore, we investigated whether the SCORE2 model [7] can also identify patients at highest risk for colorectal neoplasia by retrospectively evaluating an Austrian screening cohort. 

## 2. Methods

### 2.1. Subjects

We included participants from the Salzburg Colon Cancer Prevention Initiative (Sakkopi). The latter is a cohort of asymptomatic patients screened for CRC between January 2007 and March 2020 at a single center in Austria. The total cohort consists of 5977 consecutive patients. A total of 694 patients were excluded due to a prior history of cardiovascular disease (coronary artery disease, peripheral artery disease, transient ischemic attack, or stroke), 759 patients did not meet the age criteria for SCORE2 (40–69 years) and were therefore excluded, and 1113 further patients were excluded due to missing data for calculating SCORE2, leaving a total of 3408 patients for the final analysis.

### 2.2. Patient Assessment for Risk Factors

As previously described, patients participating in this study were examined on two consecutive days [8]. Vital signs, clinical examination, as well as a laboratory assessment were performed on the day of hospital admission, whereas colonoscopy was performed on the following day. Additionally, patients completed a questionnaire about their family and medical history. Body mass index (BMI) was defined according to the World health organization (WHO) and arterial hypertension according to the ESC guideline on the management of arterial hypertension [9]. Smoking status was categorized into “ever smokers” and “active smokers” based on the information given and the metabolic syndrome was defined according to the IDF/AHA/NHLBI consensus [10]. Additionally, family history of colorectal carcinoma (CRC) was assessed and patients with a first degree relative (parents, siblings, or children) with CRC were defined as having a positive family history.

### 2.3. Assessment of Cardiovascular Risk

The SCORE2 model, published in 2021, is used to estimate the 10-year risk of fatal and non-fatal cardiovascular disease in Europe [7]. It is intended to be used in patients aged 40–69 years without a prior history of cardiovascular disease. Risk factors included in this algorithm are sex, age, smoking status, systolic blood pressure, and total and high-density lipoprotein (HDL) cholesterol. The Stata code for the SCORE2 algorithm was requested from the study authors and calculated accordingly.

### 2.4. Assessment of Colorectal Lesions

Colonoscopy was performed according to recommendations by international guidelines and all performance measures were reached [11]. All polyps were sent for histopathologic analysis and were characterized based on their macroscopic and histologic results. Any colorectal neoplasia was defined if an adenoma, an advanced adenoma, or a CRC was found. An advanced colorectal neoplasia was present if an adenoma was (1) ≥1 cm, (2) had high-grade dysplasia, or (3) villous features [12,13], or a CRC was described histologically.

### 2.5. Mortality Data Assessment

Data on death and ICD coded causes of death were retrieved on 25 June 2021 from the Austrian “Sterberegister” based on the individual social security number of each Austrian individual.

### 2.6. Statistical Analysis

The primary endpoint was the diagnosis of any colorectal neoplasia in the screening colonoscopy. The secondary endpoints of this study were the presence of advanced colorectal neoplasia, the tumor-related mortality, and the all-cause mortality during the follow-up. We fitted models for the dependent variables “any colorectal neoplasia” or “advanced colorectal neoplasia” using multilevel logistic regression with robust standard errors with the year of inclusion as a random effect and the SCORE2 as a fixed effect (model-1). We obtained odds ratios (OR) and respective 95% confidence intervals (95%CI) for the binary endpoints. The OR describes the change in the odds of the dependent variable (any adenoma or advanced adenoma) per each unit increase for the continuous variable SCORE2 per percent point and for one specific category versus a reference category for categorical variables. The regression analyses were conducted using only robust estimators of the standard errors and not in the sense of robustness against violations of normality assumptions as for the robust methods (e.g., Mann–Whitney tests) used for the univariate analyses.

**Figure 1:**
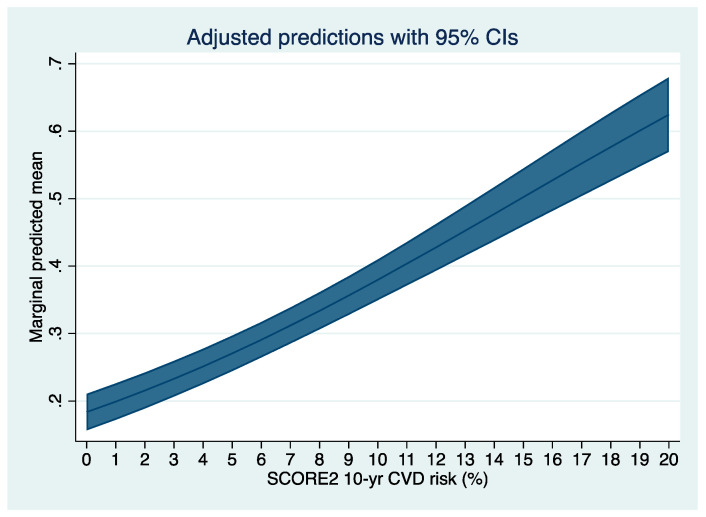
Predicted risk for any colorectal neoplasia based on the SCORE2 obtained by univariable multilevel logistic regression, using the year of inclusion as random effect and the SCORE2 as continuous variable as fixed effect.

**Figure 2:**
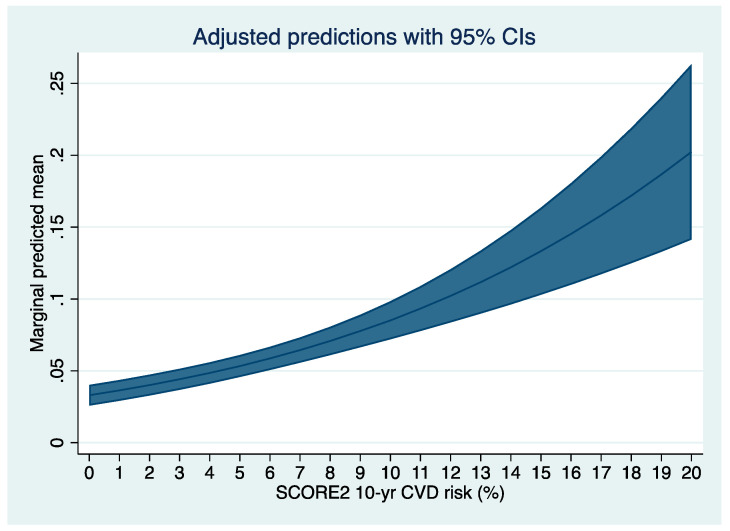
Predicted risk for any advanced colorectal neoplasia based on the SCORE2 obtained by univariable multilevel logistic regression, using the year of inclusion as random effect and the SCORE2 as continuous variable as fixed effect.

**Figure 3:**
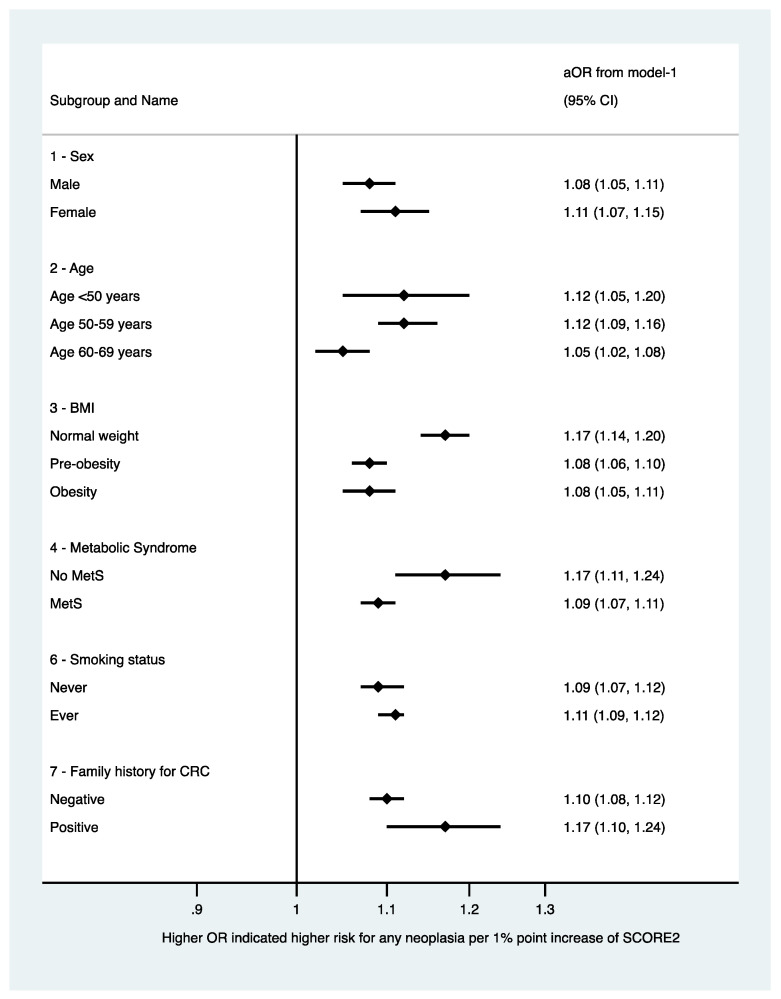
Sensitivity analyses stratifying the presence of the primary endpoint (any colorectal neoplasia) according to patient-specific baseline characteristics (stratified for sex, age (in categories), BMI (in categories according to the World Health Organization), smoking status, metabolic syndrome, and positive family history). For the sensitivity analyses, model-1 was fitted with SCORE2 as continuous variable as independent variable and any colorectal neoplasia as dependent variable in the strata. Abbreviations: BMI: body mass index; CI: confidence interval; and OR: odds ratio.

**Figure 4:**
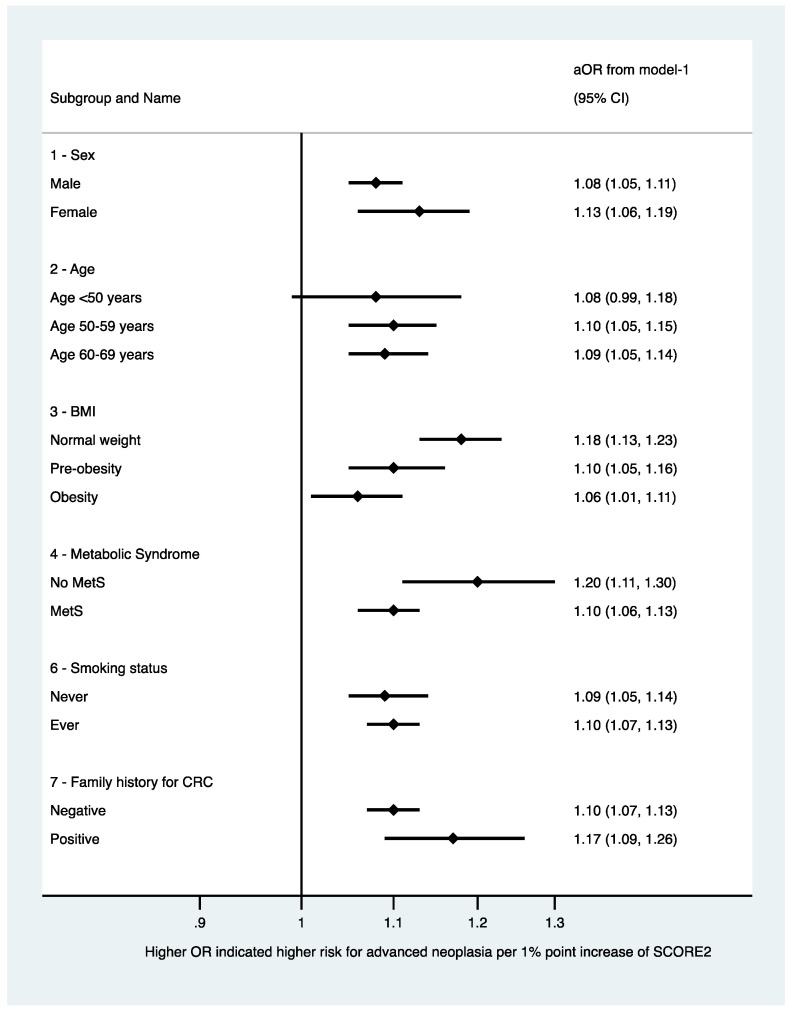
Sensitivity analyses stratifying the presence of the primary endpoint (any colorectal neoplasia) according to patient-specific baseline characteristics (stratified for sex, age (in categories), BMI (in categories according to the World Health Organization), smoking status, metabolic syndrome, and positive family history). For the sensitivity analyses, model-1 was fitted with SCORE2 as continuous variable as independent variable and advanced colorectal neoplasia as dependent variable in the strata. We plotted the OR and 95%CI. Abbreviations: BMI: body mass index; CI: confidence interval; and OR: odds ratio.

**Figure 5:**
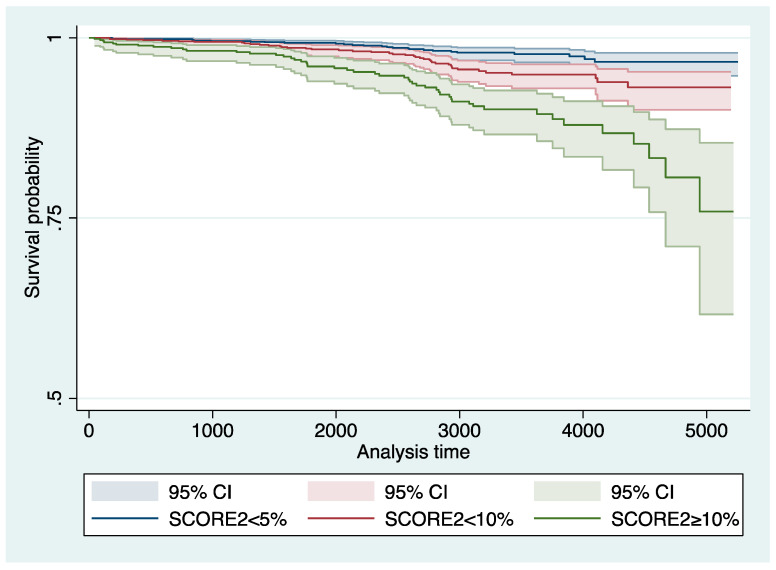
Survival data for all-cause mortality in the three SCORE2 strata. The all-cause mortality was 7% over a median follow-up of 2768 days. In the Cox regression model, SCORE2 was associated with all-cause (HR 1.11 95%CI 1.09–1.14; *p* < 0.001) mortality. Abbreviations: CI: confidence interval; and HR: hazard ratio.

We plotted the predicted risk for any colorectal neoplasia based on the SCORE2 obtained by multilevel logistic regression in Figure 1 and for any advanced colorectal neoplasia in Figure 2. Further, we fitted a multivariable multilevel logistic regression model with the presence of any or an advanced colorectal neoplasia as dependent variable, the year of inclusion as random effect, the SCORE2, age, sex, a family history of first-degree relatives for CRC, and the concomitant diagnosis of metabolic syndrome as fixed effects (model-2). We chose the covariables based on our own clinical experience and previous literature. We further decided to adjust for presence of metabolic syndrome, as its diagnostic criteria are not congruent with the variables included in the SCORE2 to avoid (further) overfitting considering the variables included in the calculation of SCORE2. We chose to adjust for age and sex although these variables are included in the SCORE2 based on our clinical experience and evidence under striking the importance of age and sex in both cardiovascular and oncological risk [14,15,16]. We performed sensitivity analyses stratifying the presence of the primary endpoint (any colorectal neoplasia) according to patient-specific baseline characteristics: We stratified for sex, age (in categories), BMI (in categories according to the WHO definition), smoking status, metabolic syndrome, and positive family history. For the sensitivity analyses, we fitted model-1 with SCORE2 as continuous variable as independent variable (fixed effect) and any adenoma or advanced colorectal neoplasia as dependent variable in the groups. We plotted the OR and 95%CI of the sensitivity analyses in forest plots (Figure 3 and Figure 4). Further, we assessed the association between SCORE2 and the mortality endpoints (tumor-related and all-cause mortality) using proportional hazard Cox regression and obtained hazard ratios (HR) and respective 95% confidence intervals (95%CI). The HR describes the change in risk of death per each unit increase for the continuous variable SCORE2 per % and for one specific category versus a reference category for categorical variables. We plotted the survival data using a Kaplan–Meier curve in Figure 5. Continuous data are given as median ± inter-quartile range (IQR) and compared using Mann–Whitney U-Test or mean ± standard deviation (SD) and compared using Student’s *t*-test accordingly. Categorical data are given as numbers (percentage) and compared using the chi-square test. All tests were two-sided, and a *p*-value of <0.05 was considered statistically significant. Stata/IC 17 was used for all statistical analyses.

### 2.7. Ethics Statement

We performed the study and all procedures according to the principles of the Declaration of Helsinki. The local ethics committee for the province Salzburg approved the study protocol (approval no. 415-E/1262). Written informed consent was obtained from every participant.

## 3. Results

The adenoma detection rate in 3408 patients undergoing colonoscopy was 31%, the mean adenoma detection rate was 0.53 per colonoscopy, and the cecum intubation rate was 98.8%.

### 3.1. Baseline Demographics in the SCORE2 Strata

Patients in the SCORE2 > 10% stratum were less often female (22% vs. 66%; *p* < 0.001), older (62 ± 6 vs. 52 ± 5; *p* < 0.001), had a higher BMI (29 ± 5 vs. 26 ± 5; *p* < 0.001), and more frequently had metabolic syndrome (93% vs. 64%; *p* < 0.001) than patients in the low-risk SCORE2 stratum with a SCORE2 risk of <5% (Table 1).

### 3.2. Association between SCORE2 and Colorectal Adenoma

The rates of colorectal neoplasia were 20%, 37%, and 44% in the three SCORE2 strata. Similarly, the rate of advanced colorectal neoplasia increased from 4% to 7% and 13% across the SCORE2 strata. As a continuous variable, SCORE2 was associated with the presence of any colorectal neoplasia (OR 1.16 95%CI 1.09–1.12; *p* < 0.001) and advanced colorectal neoplasia (OR 1.11 95%CI 1.08–1.14; *p* < 0.001) in univariable multilevel logistic regression. In Figure 1 we plotted the predicted risk for any colorectal neoplasia based on the SCORE2 and in Figure 2 for advanced colorectal neoplasia.

After multivariable adjustment for age, sex, and the concomitant diagnosis of metabolic syndrome, a higher SCORE2 remained associated with higher odds for both any (aOR 1.04 95%CI 1.01–1.07; *p* = 0.01) and advanced (aOR 1.06 95%CI 1.03–1.11; *p* = 0.001) colorectal neoplasia. In Table 2 we further show the localization of colorectal neoplasia in the SCORE2 strata. We found that that the amounts of proximal, distal, and rectal colorectal neoplasia increased with a higher SCORE2 (Table 2).

### 3.3. Sensitivity Analyses

We fitted logistic regression models with SCORE2 as the independent variable and any colorectal neoplasia (Figure 3) and advanced colorectal neoplasia (Figure 4) as the dependent variable in patient-specific strata for sensitivity analyses. A higher SCORE2 was associated with higher odds for both any colorectal neoplasia as well as advanced colorectal neoplasia in both sexes, all age and BMI categories, regardless of the smoking status and the presence of metabolic syndrome.

### 3.4. Association between SCORE2 and Mortality

The all-cause mortality was 7% and the tumor-related mortality was 2% over a median follow-up of 93 months. In the Cox regression model, SCORE2 was associated with both all-cause (HR 1.11 95%CI 1.09–1.14; *p* < 0.001), tumor-related (HR 1.10 95%CI 1.05–1.14; *p* < 0.001), and cardiovascular (HR 1.12 95%CI 1.06–1.19; *p* < 0.001) mortality. All-cause mortality was 2%, 3%, and 8% (*p* < 0.001) in the three SCORE2 strata and the tumor-related mortality was 1% in the two lower SCORE2 strata and 2% in patients with SCORE2 ≥ 10% (*p* = 0.08). We plotted the survival data for all-cause mortality in the three SCORE2 strata in Figure 5.

## 4. Discussion

Our study shows that the SCORE2 model validated for estimating the 10-year fatal and non-fatal cardiovascular risk also predicts the detection of colorectal neoplasia and advanced colorectal neoplasia. Patients with the highest SCORE2 levels are at the highest risk for colorectal neoplasia. Additionally, all-cause mortality and tumor related mortality increase with a rising SCORE2 level.

While the prediction of cardiovascular disease risk and consecutive treatment of cardiovascular disease risk factors has not been shown to reduce mortality rates [6], CRC screening can effectively reduce CRC mortality [17]. However, low participation rates in both opportunistic, but also in systematic screening remains a challenge. In a CRC screening study of 53,000 Spanish patients, only 34% participated in screening via a stool test and even less patients in the screening via the colonoscopy group (25% participation rate) 5.

We assume that a direct and personal invitation by the general practitioner in charge could be another useful measure to increase the participation rate. A tool that could be used to assess the individual risk of a patient and communicate it accordingly could also be particularly effective. Cardiology societies have developed successful scoring tools to calculate the risk of a cardiovascular event. There are various cardiovascular risk calculators that are available, however, only some of them are externally validated [18].

The Framingham risk score was the first score established in 1998 [19] and is based on an U.S. American cohort of 5000 individuals. Limitations of this score are that it is validated only in American Caucasians and only predicts future coronary heart disease. It does not include other cardiovascular events such as stroke, transient ischemic attack, and heart failure [20,21]. The American College of Cardiology (ACC) and the American Heart Association (AHA) consecutively published the ASCVD (Atherosclerotic Cardiovascular Disease) Risk Calculator in 2013 [21]. This score was calculated based on a pooled cohort of participants of different large cohort studies, including the Cardiovascular Health Study [22], the Framingham Original and Offspring Study cohort [23,24], the ARIC (Atherosclerosis Risk in Communities study) [25], and the CARDIA (Coronary Artery Risk Development in Young Adults) study [26]. Therefore, this score is considered more valid for the entire US population and is validated to estimate the 10-year risk of a first atherosclerotic event, including non-fatal myocardial infarction, coronary artery disease, and fatal or non-fatal stroke [21]. Variables included in this score are age, total cholesterol, HDL cholesterol, systolic blood pressure, diabetes mellitus, and current smoking status [21]. The ESC published its own score in 2003. The SCORE pooled datasets from 12 European cohort studies of around 205,000 persons and estimates the 10-year risk of fatal cardiovascular disease [27]. It is validated for persons aged 45–64 and includes age, sex, current smoking status, total cholesterol and HDL cholesterol, and systolic blood pressure. This score was only recently updated because SCORE only considers fatal CVD outcomes and underestimates the total CVD burden. It was also based on cohorts recruited before 1986, which do not represent current CVD rates [7]. The new SCORE2 estimates the 10-year fatal and non-fatal CVD risk in patients without previous CVD aged 40–69 years.

Smaller studies investigated whether these risk scores could also predict the risk of other diseases or death. The ASCVD score was shown to be independently associated with the increased risk of future cancer [28]. Additionally, the ASCVD score was able to predict cardiovascular and all-cause mortality [29]. Furthermore, the Framingham Risk Score was associated with colorectal neoplasia [30].

The robust association of cardiovascular and CRC risk could be due to common risk factors: obesity, diabetes, dyslipidemia, and hypertension are associated with increased inflammatory cytokines and chronic inflammation, which can not only lead to cardiovascular death but have also been shown to be involved in the development of colorectal neoplasia and colorectal carcinomas [31]. Cardiovascular and oncological diseases also share risk factors such as dietary errors, a predominantly sedentary lifestyle, and smoking.

As SCORE2 is now the most recent tool for predicting cardiovascular events, we investigated the relationship between SCORE2 and colorectal neoplasia. We found a robust association between cardiovascular risk and the likelihood of finding a colorectal neoplasia during screening colonoscopy. We were able to confirm this association in extensive sensitivity analyses for both sexes, all included age groups and patients with and without a positive family history for CRC. SCORE2 was also associated not only with all-cause but also with tumor-related mortality, which we believe underlines the association of SCORE2 with non-specific oncological risk. We therefore think that SCORE2 should, on the one hand, remind clinicians that patients with an unfavorable cardiovascular risk profile should be particularly encouraged to undergo screening colonoscopy. On the other hand, SCORE2 could also be used to communicate these two risks (cardiovascular and oncological) effectively to patients.

This study has several limitations. First, SCORE2 was only recently developed and therefore, only retrospectively calculated for the SAKKOPI collective. Therefore, this is a retrospective post-hoc study. Second, we consider this study to be primarily cross-sectional, and the follow-up thesis-generating. This is because the mortality data were obtained from the Austrian “death register”, which, although complete, is only very imprecise regarding the cause of death. Especially in view of decreasing autopsy rates, the exact cause of death is not always ascertainable. Furthermore, not all tumor-related deaths were caused by colorectal carcinoma. Thus, we cannot completely exclude the possibility that other carcinomas contributed to our results.

Nevertheless, we think that this study also has specific strengths. We can access granular cardiometabolic data from a large collective of asymptomatic patients who underwent screening colonoscopy and were therefore able to calculate SCORE2 post-hoc and, in our opinion, demonstrate in this study that SCORE2 predicts not only cardiovascular risk but also risk for colorectal neoplasia. Since we assume that the ESC will invest resources in promoting the use of SCORE2, we put up for debate whether it would be useful in the sense of a united appearance if gastroenterological societies again emphasize the value of screening colonoscopies, especially for patients at high cardiovascular risk and use SCORE2 to promote this clinically important issue.

## 5. Conclusions

We believe that due to the increasing cost pressure on healthcare systems worldwide, but especially from the patients’ point of view, disease prevention should be a scientific and clinical focus. We think that it should be the task of medical societies to provide simple and pragmatic tools for the clinician. The SCORE2 was developed to estimate cardiovascular risk, but in the present study it was also associated with a risk for colorectal neoplasia. We therefore think that SCORE2 could be used in clinical practice to approximate the probability of colorectal neoplasia. An intellectual linkage of SCORE2 could therefore remind clinicians to work with patients on the mitigation of cardiovascular and oncological risk.

## Figures and Tables

**Table 1:** Baseline characteristics of patients according to their SCORE2 level (group 1: cardiovascular risk < 5%, group 2: CV risk between 5 and 9.9%, and group 3: CV risk ≥ 10%). Abbreviations: CV: cardiovascular.

	SCORE2 < 5%	SCORE2 5–9.9%	SCORE2 ≥ 10%	*p*-Value
	N = 1537	N = 1235	N = 636	
Sex				<0.001
Male, % (*n*)	34% (527)	65% (803)	78% (496)	
Female, % (*n*)	66% (1010)	35% (432)	22% (140)	
Age (years)	52 (5)	58 (6)	62 (6)	<0.001
Age categories				<0.001
Age < 50 years, % (*n*)	28% (435)	7% (88)	3% (17)	
Age 50–59 years, % (*n*)	64% (976)	52% (641)	30% (180)	
Age 60–69 years, % (*n*)	8% (126)	41% (497)	68% (412)	
BMI	26 (5)	28 (4)	29 (5)	<0.001
BMI categories				<0.001
Underweight, % (*n*)	1% (16)	0% (4)	0% (1)	
Normal weight, % (*n*)	50% (765)	28% (344)	20% (126)	
Pre–obesity, % (*n*)	34% (523)	47% (586)	44% (278)	
Obesity, % (*n*)	15% (233)	24% (301)	36% (231)	
Systolic BP (mmHg)	124 (15)	135 (16)	147 (20)	<0.001
Diastolic BP (mmHg)	78 (9)	82 (9)	85 (11)	<0.001
Arterial hypertension, % (*n*)	32% (493)	62% (762)	83% (527)	<0.001
Current smoker, % (*n*)	16% (253)	33% (403)	45% (289)	<0.001
Ever smoker, % (*n*)	62% (952)	72% (885)	76% (483)	<0.001
Cholesterol, (mg/dL)	222 (39)	229 (44)	223 (49)	<0.001
LDL (mg/dL)	140 (36)	150 (40)	147 (43)	<0.001
HDL (mg/dL)	64 (17)	55 (14)	50 (13)	<0.001
Triglycerides (mg/dL)	105 (54)	141 (100)	171 (137)	<0.001
CRP (mg/dL)	0.3 (0.7)	0.3 (0.5)	0.4 (0.8)	<0.001
HbA1c (%)	5.4 (0.4)	5.5 (0.4)	5.9 (0.8)	<0.001
Fasting glucose (mg/dL)	94 (10)	101 (20)	118 (43)	<0.001
Metabolic syndrome, % (*n*)	64% (980)	84% (1042)	93% (594)	<0.001

**Table 2:** Any colorectal neoplasia and advanced colorectal neoplasia detection rates according to SCORE2 level. Abbreviation: NNS—number needed to screen.

	SCORE2 < 5%	SCORE2 5–9.9%	SCORE2 ≥ 10%	*p*-Value
	N = 1537	N = 1235	N = 636	
Any neoplasia	20% (313)	37% (463)	44% (281)	<0.001
NNS	5	3	2	
Mean adenoma detection rate	0.28 (0.67)	0.60 (0.97)	0.96 (1.61)	<0.001
Number of neoplasia				<0.001
0	80% (1224)	63% (772)	56% (355)	
1	15% (233)	23% (283)	22% (143)	
2	3% (52)	9% (115)	8% (51)	
3	1% (19)	4% (44)	7% (43)	
4	0% (6)	1% (12)	3% (22)	
5	0% (2)	0% (6)	1% (8)	
6	0% (0)	0% (2)	1% (5)	
7	0% (1)	0% (1)	0% (1)	
8	0% (0)	0% (0)	1% (5)	
9	0% (0)	0% (0)	0% (1)	
≥10	0% (0)	0% (0)	0% (2)	
Neoplasia in proximal colon, % (*n*)	12% (189)	23% (289)	30% (192)	<0.001
Neoplasia in distal colon, % (*n*)	8% (128)	17% (210)	21% (135)	<0.001
Neoplasia in rectum, % (*n*)	3% (48)	5% (63)	7% (41)	0.001
Advanced neoplasia, % (*n*)	4% (59)	7% (86)	13% (80)	<0.001

## Data Availability

Data are available upon reasonable request from the corresponding author.
